# Psychological Intrusion – An Overlooked Aspect of Dental Fear

**DOI:** 10.3389/fpsyg.2018.00501

**Published:** 2018-04-17

**Authors:** Helen R. Chapman, Nick Kirby-Turner

**Affiliations:** ^1^School of Psychology, University of Lincoln, Lincoln, United Kingdom; ^2^Retired, West Sussex, United Kingdom

**Keywords:** intrusion, intrusive parenting, psychological control, dental fear, dental anxiety, parenting, temperament

## Abstract

Dental fear/anxiety is a widely recognised problem affecting a large proportion of the population. It can result in avoidance and/or difficulty accepting dental care. We believe that psychological intrusion may play a role in the aetiology and maintenance of dental fear for at least some individuals. In this narrative review we will take a developmental perspective in order to understand its impact across the lifespan. We will consider the nature of ‘self,’ parenting styles, the details of intrusive parenting or parental psychological control, and briefly touch upon child temperament and parental anxiety. Finally, we draw together the supporting (largely unrecognised) evidence available in the dental literature. We illustrate the paper with clinical examples and discuss possibly effective ways of addressing the problem. We conclude that psychological intrusion appears to play an important role in dental fear, for at least some individuals, and we call for detailed research into the extent and exact nature of the problem. A simple means of identifying individuals who are vulnerable to psychological intrusion would be useful for dentists.

## Introduction

Dental fear/anxiety is a widely recognised problem affecting a large proportion of the population, resulting in avoidance and/or difficultly in accepting dental care (Humphris et al., 2013; Armfield and Ketting, 2015). Two recent reviews of the field (Carter et al., 2014; Seligman et al., 2017) failed to identify any evidence for psychological intrusion being associated with the aetiology, maintenance or treatment of dental fear. Armfield (2011), Carrillo-Diaz et al. (2012), and Crego et al. (2013) have proposed a ‘cognitive vulnerability model’ of dental fear which references our model of dental fear (Chapman and Kirby-Turner, 1999), suggesting that relevant threat cognitions should be identifiable. However, we would argue that the process of psychological intrusion is insidious and will be largely unidentifiable by patients, except as a feeling of unease, though ‘clues’ may be offered to the alert practitioner.

This brief, narrative review of psychological intrusion or control will take a developmental perspective. To understand psychological intrusion in adulthood, it is necessary to first understand the development and impact of psychological intrusion in childhood and the vulnerabilities thus created in the individual. It will consider the nature of ‘self,’ parenting styles, the details of intrusive parenting or parental psychological control, briefly touch upon child temperament and parental anxiety and then consider how psychological intrusion or control may impact the individual across the lifespan.

Finally, we draw together the supporting (largely unrecognised) evidence available in the dental literature. We illustrate the paper with clinical examples and discuss possibly effective ways of addressing the problem.

In writing the paper we were aware of the conflicting needs of readers who are psychologists, and therefore well informed regarding the psychological issues covered, and readers with a dental background who may be less familiar with some or all of the issues covered. The paper seeks to address this.

## The Nature of the Self

Individuals have a conscious model of the ‘self’ and of identity as ‘a self as one experiences oneself’ (Dagnan et al., 2002), that is it is only accessible to oneself. This is continually remodelled through social interactions which re/construct one’s core self-schema (Beck et al., 1979). A healthy inner model is achieved through the validation, by others of an outer, public self. This construction can be conscious or unconscious, but is the desired self (Dagnan et al., 2002).

**FIGURE 1:**
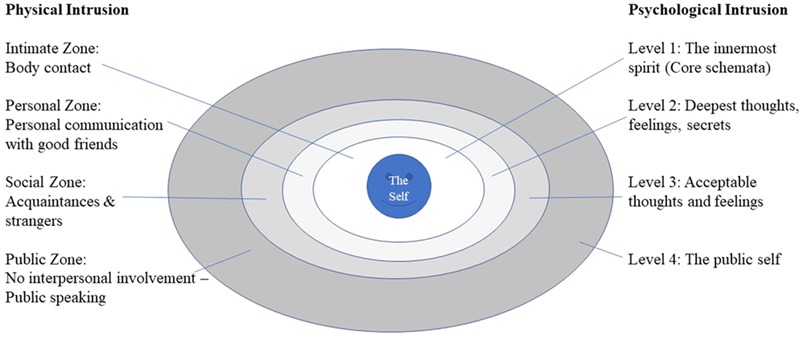
The four depths of psychological self and interpersonal space adapted from Scott (1993), with permission.

This ‘psychological self,’ has been conceptualised as four ‘depths’ of self, (Scott, 1993; Geanellos, 2003) similar to the layers of physical interpersonal space (**Figure 1**):

• Level 1 – an inner core – core schemata
• Level 2 – deepest thoughts, feelings and secrets
• Level 3 – thoughts and feelings perceived as generally acceptable
• Level 4 – outermost layer - the public self.

In adulthood, the ‘self’ is vulnerable to exclusion and distance which result in insecurity, seeking to please and fear of failure to do so. It is also vulnerable to intrusion and control (Dagnan et al., 2002).

An intrusive ‘assault’ may leave the individual feeling coerced into presenting a different self to the world (Level 4) or, at a deeper level, that their core (Level 1) is being defined by another with a loss of autonomy, where another defines one’s sense of self (Dagnan et al., 2002). There may be a major fear of being ‘taken over’ by another. This often results in shame (Dagnan et al., 1998). Dental patients for whom this is relevant will be vulnerable to any sense of loss of control or threat to their ‘self’ or self-esteem at any of the 4 levels of psychological intrusion. In a broader context, intrusion is measured by the Self and Other Scale (Dexter-Smith et al., 2003; pp. 102–103) which includes items such as, ‘I dread being under someone else’s control.’ and ‘Often I wish people would give me space to be myself,’ which give a sense of how vulnerability in the dental clinic may arise.

We believe psychological assaults on the self can also happen to children at all four levels within the dental environment, though the exact ‘depth’ of danger will depend on the meaning of the assault to the patient. For example, the dentist runs a prophylaxis brush over a 6-year-old’s teeth. (He has gingivitis, so it bleeds as the dentist accidently clips the gum; it may be uncomfortable.) The child says, ‘That hurts.’ To which the dentist (and sometimes the mother) reply, ‘Oh, no it doesn’t. It can’t. It’s just an electric toothbrush.’ For some children, this will simply be taken as a denial of their painful experience (most likely impacting at Level 3; “It really was uncomfortable, no matter what you say.”) For others, (and this probably includes those where the mother joins in) this is imposing another’s sense of reality on the experience and will be intrusive at a deeper level, as, ‘My experience is wrong/unheard’ (“But I told you it hurts! Why don’t you believe me?”; Level 2) or, “I am such a wimp” (Level 1). Similarly, dentists are trained to prepare the patient for sensations/discomfort/pain using language that tends to diminish perceived sensation as a way of reducing fear. However, the child may experience this as an enormous, ‘Ouch.’ If this discrepancy is about a prophylaxis brush on an incisal edge, then reconstructing of pain scales is required (Chapman and Kirby-Turner, 2002, 2005) but if it is about a deep cavity with a local anaesthetic in place, it should never be denied as this is overtly intrusive. It is possible to imagine parallel experiences for adult patients. Wepman and Sonnenberg (1978) noted that dentists sometimes tried to shame children into changing their behaviour.

## Parenting Style

Parenting styles (Baumrind, 1966; Baumrind, 2012) may be categorised as

1. *authoritarian* (high demand/low responsiveness; controlling, restrictive, disciplinarian approach, setting high standards with poor emotional support)
2. *authoritative or ‘good enough’* (emotionally supportive environment where reasonable standards are set and expected consistently, with appropriate granting of autonomy fostering independence and development)
3. *permissive or ‘laissez faire’* (low demand/high responsiveness; few demands are made, little discipline enforced and low levels of control imposed). Permissive parenting may be divided to create a neglectful group (low demand/low responsiveness) (Maccoby and Martin, 1983). Baumrind (2012) emphasised that both authoritarian and authoritative parenting are power-assertive, but it is the coercive nature of authoritarian parenting that is detrimental.

Self-reported parenting style (Robinson et al., 1995) is associated with internalising and externalising disorders in children (Berg-Nielsen et al., 2002) and has been found to influence dentist–child–parent interactions in the dental clinic (ten Berge et al., 2003; Aminabadi and Farahani, 2008; Armfield, 2010; Krikken et al., 2012a; Aminabadi et al., 2015), though this is not a consistent finding (Krikken and Veerkamp, 2008; Krikken et al., 2012a, 2013). Compared to permissive and authoritarian parenting, authoritative parenting is associated with ‘more positive’ behaviour in the dental clinic (Howenstein et al., 2015).

Simplifying parenting constructs, Barber and colleagues (Barber and Harmon, 2002; Barber et al., 2002) consider two categories of parenting:

• *parental support* (a unidimensional construct encompassing behaviours such as nurturing, warmth, responsiveness, and acceptance).
• *parental control* (behavioural and coercive psychological control or intrusion, which is discussed in more detail below). Psychological control interferes with a child’s ability to become independent, to develop a healthy sense of self and personal identity whilst *appropriate* behavioural control facilitates development by providing supervision and guidance (Smetana and Daddis, 2002).
• *parental support* relates to positive child development; maternal warmth can neutralise the negative impact of intrusive parental behaviour (Raudino et al., 2013). At least in early childhood, it may be that mothers’ parenting impacts more on daughters than sons (Ollendick and Horsch, 2007; Barnett and Scaramella, 2017).

Possibly because psychological control is such a complex construct, it has been found to be a separate construct from ‘autonomy granting’; they do not form either end of a continuum of parenting (Silk et al., 2003). The relationship, in children, between low levels of maternal autonomy granting and increased emotional reactivity and the emotion regulation strategies used, is moderated by child perceived control (Benoit Allen et al., 2016).

Parents and children both seek to control the private aspects of the child’s life such as preferences and choices of friends, activities, the state of his/her body and privacy. There is an age-related decrease in the areas perceived as legitimate for parental control and conflict between adolescents and their parents as to where the boundaries lie. Adolescents generally see parent control over moral and conventional issues as legitimate. However, if parents try to control personal issues, they are perceived as psychologically controlling (Smetana and Daddis, 2002). A dentist who is being too forthright regarding the need to clean one’s teeth may find her/himself similarly perceived. We have noticed that the flaunting of unhealthy eating habits (for example sugary snacks and drinks between meals) in defiance of professional advice and parental wishes, appears to be one of the few areas in which some children can seize control.

A large study of adolescents (Young et al., 2011) found that children’s *perceptions* of emotional neglect and control were not associated with objective measures of neglect and control and were independently associated with later psychiatric disorders. This reflects the problem for dentists; one child’s reassurance is another child’s intrusive threat, presumably based on the intrusiveness of their learning experiences within the family. For dentists this is a particular issue; non-cooperative behaviour may be interpreted as non-compliance and may result in intrusive coercion and threats by the dentist, or fear, which is likely to result in encouragement and reassurance (see below). These latter dentist behaviours are usually associated with reduced fear-related child behaviours, but may, apparently counter-intuitively, escalate the situation. This may partly be due to psychological intrusion. As we wrote this paper, NK-T ‘confessed’ to HC that, initially in their joint clinics, he had had to get HC to pause and get her to give the children a chance to think things through. Even as a dentist who was skilled in treating frightened children, she still had a tendency to ‘leap in and reassure and encourage,’ with a tendency to overwhelm the most vulnerable. Dentists are trained to problem solve and intervene; sometimes they need someone to say, ‘let’s pause here and find out a little more’ before they are unwittingly guilty of being intrusive like the parent.

More recently identified styles of ‘overparenting’ have been identified including the ‘hyper-parenting’ styles (Segrin et al., 2013; Janssen, 2015) of:

• *“helicopter parents”* who try to protect their child from all dangers and to solve all of his/her problems.
• *“little emperor”* parents who strive to give their children all the material goods they desire.
• *“tiger moms”* who push for and accept nothing less than exceptional achievement from their children. For example, HC once treated a 6-year-old who was undoubtedly intelligent, but claimed to have read the entirety of *The Hobbit* in 4 days.
• *“concerted cultivation”* the scheduling of several extracurricular activities to provide them with an advantage.

Overparenting includes risk aversion, a preoccupation with the child’s happiness, and the drive to pre-emptively solve the child’s problems. It is driven by parents’ over enthusiastic wish to ensure the success and happiness (defined in parental terms) of their child, by removing any obstacles along the way. It may represent enmeshment (Segrin et al., 2012) (see below).

## Parental Psychological Control or Intrusive Parenting

The characteristics of parental psychological control or intrusion fall into 5 main categories (Barber and Harmon, 2002):

(1) In manipulative behaviour, the parent (usually the mother) attempts to manipulate the child’s behaviour and/or shift the emotional balance within the relationship by a variety of means, as illustrated by examples from HC’s dental clinic:
  • *Guilt induction/blaming* – “You’ve got to have it done now– I’ve taken the morning off work to bring you” (Nelson and Crick, 2002).
  • *Contingent affection* – parental affection and attention are contingent on the child being or behaving as the parent would like. “I’m not going to hold your hand unless you behave.”
  • *Trying to change how the child thinks*. “Are you sure it feels OK?” (I wouldn’t like it!) (Nelson and Crick, 2002).
  • *Instilling anxiety*, although this has been excluded from more recent measures of psychological control. “You wouldn’t catch me sitting in that dental chair” or “I don’t like the dentist.”
  • *Excluding outside influence -* “I don’t care what Mrs Smith (class teacher) at school told you, she doesn’t know anything.”
  • *Being unfriendly* when the child has a different point of view to the parent. “I know you don’t want to have the fissure sealants, but is that the way to pay me back for all the effort I’ve taken bringing you here” (Nelson and Crick, 2002).
  • *Expressions of disappointment/excessive criticism*, which may be accompanied by a refusal to talk to the child. “I’m *really* upset with you. I thought you’d be braver than that” (Nelson and Crick, 2002).
  • *Shaming* and laughing at child’s mistakes despite fact that child is trying very hard (Sroufe et al., 1993). “Your little brother is braver than you are.” Dentists can compound this by using a more confident, younger sibling as a role model.
  • *Bringing up past mistakes during criticism*. “Oh, you are so difficult at the dentist; it’s just the same as you being difficult going to the doctor” (Nelson and Crick, 2002).
  • *Invalidation/ignoring of feelings*. “Don’t be silly, that doesn’t hurt.”
  • *Derision* (derogatory comments, sarcasm and scolding), especially in the company of others (Rubin et al., 2002). “Oh, don’t bother the dentist with such a silly question.” “Oh, come on Lucy, you’ve never managed to do it before, why do you think you can do it now?”

It is interesting to note how many of these scenarios are shame-inducing and may well feed into the model of the self which is described above.

(2) In constraining/binding behaviour, the child’s self-expression of his/her anxieties, conflicts and disagreements with parental rules is actively discouraged or limited to parental interests, thus undermining the child. “I know you don’t like the toothpaste, but that’s the one we buy because your Dad likes it.” “And please don’t start on about how you don’t want to miss football to come here – you are simply not missing lessons.” Self-expression and discovery are inhibited as the child is undermined in family interactions. Reflective learning opportunities about the self and others are lost.
(3) Expectations and achievement demands of the child are excessive and standards set are absolute. “He can’t come to an appointment then as he has to practise for his clarinet exam and we’re hoping he’ll do really well – he needs to get a distinction so that he can get a scholarship to college.”
(4) The parent may oscillate between caring and over-involvement to attacking behaviours. The parent may behave as a peer of the child or may defer to the child for direction. This confuses the child about his/her acceptability to the parent and thus maintains parental power (Sroufe et al., 1993). It may be associated with disorganised attachment (Wang et al., 2015).
(5) Parents are possessive; they may ‘baby’ the child, encourage psychological and emotional dependence, unduly emphasise the affectional bonds between the parent and child and limit the child to activities within the family realm (Wood, 2006). This overprotection can take several forms; age inappropriate and unnecessary help with self-help tasks such as dressing, which the child is capable of carrying out; use of baby words/language; excessive physical affection; invasions of privacy. These are intrusive because they oblige the child to function at an immature level (Wood et al., 2007). They include, for young children, sitting the child on the lap or initiating physical affection, such as kissing or caressing *before* a task is complete, i.e., it is distracting from the task which actually requires the child’s full attention (Wood, 2006). With older children, behaviours include not allowing the child to make decisions such as what to wear, encouraging dependency and restricting freedom (Parker et al., 1979). Overprotection appears to be particularly damaging for boys (Enns et al., 2002). HC has treated several only sons of much older parents who seem, to her, to be particularly vulnerable to infantilization.

Such inappropriate parental power is ensured by the child being totally subordinate to the parent by;

• having to conform to parental wishes (the mother who tries to insist that her reluctant teenager has orthodontic treatment, even though he doesn’t want it)
• seeking permission for everything (the child who looks to his mother before he is prepared to accept the proffered sticker for good behaviour)
• the parent attempting to shape the behaviour and attitude of the child according to an absolute standard of behaviour: “Come on Henry, you know I expect better of you than this. If you don’t do it I’ll throw away your bicycle [a birthday present].”

It can thus be seen that parental psychological control or intrusive parenting can impact the child’s experience in the dental clinic in many ways.

At its most corrosive, the possessiveness and dominance are so severe that there is a process of enmeshment in which there is a blurring of psychological boundaries in favour of a family identity (Barber and Buehler, 1996; Kivisto et al., 2015). Children from enmeshed families have increased levels of anxiety, mediated by their temperament and sensitivity to threat, their psychological flexibility and self-compassion (Rowsell et al., 2016; Berryhill et al., 2018).

Extremely intrusive and controlling parenting can be viewed, at its extreme, as child emotional abuse (CEA) (Bell and Higgins, 2015). However, it may be limited to verbal abuse, hostility, criticism and expressions of overt dislike of the child. It damages immediately, or gradually, the behavioural, affective and cognitive functioning of the child (Sanders and Becker-Lausen, 1995).

Psychological control is thus a covert, passive-aggressive and insidious form of control that is intrusive and manipulative of a child’s thoughts, feelings, and attachment to his/her parents (Walling et al., 2007). It reflects an underlying lack of sensitivity to the child’s needs by parent(s) who are relatively uninvolved with their children (Pettit and Laird, 2002). High levels of parental psychological control are consistently associated with internalising (anxiety and depression) and externalising (delinquency, aggression, antisocial behaviour and defiance) problems in children and adolescents. This is found across cultures and age groups (Olsen et al., 2002). Both fathers and mothers (the more thoroughly researched parent) can adopt this pattern of child rearing, the level of impact on the child varying with the type of parent–child dyad and the age of the child (Stone et al., 2002).

Overcontrol may reduce a child’s opportunities to learn, his sense of mastery and competence; the child’s self-efficacy is undermined (Barber and Harmon, 2002) and s/he becomes anxious when faced with a novel task (Affrunti and Ginsburg, 2012). S/he then goes on to be anxious when faced with separation. For the dental team, this forms the difficult vicious circle of an anxious child with a ‘difficult,’ intrusive parent, whom the team would like to exclude; however, that would increase the child’s anxiety.

Constant exposure to criticism and derision results in the development of negative schema and beliefs about the self and the child may withdraw from the social world (Rubin et al., 2002). This can be detrimental to the child’s personal and social development and is linked to adjustment problems in both children and adolescents.

**FIGURE 2:**
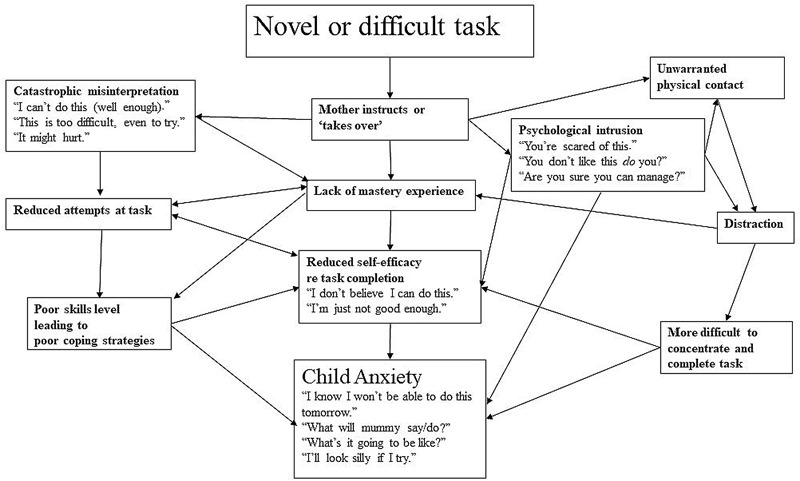
A schematic of the role of intrusive parenting in child anxiety.

We have summarised this process in **Figure 2**.

## Child Temperament

Temperament refers to children’s behavioural style, or the manner in which they interact with their environment. Temperament has been quantified into nine temperament categories (including approach and withdrawal/avoidance) and five temperament constellations (easy, intermediate low, slow to warm up, intermediate high, difficult) (Chess and Thomas, 1986). More simply, these are categorised as inhibited who are restrained, cautious and avoidant of unfamiliar people, events, and places, and uninhibited children who display spontaneous approach to novelty (Kagan et al., 1994, 2007). Inhibited children are anxiety-prone, (Kagan, 1997; Rapee, 2002; Vervoort et al., 2010) and tend to remain so through adolescence and into adulthood (Schwartz et al., 2003). In young children, intrusive control is associated with behavioural inhibition (BI), predicting its stability and moderating the risk of anxiety in children with high BI (Chronis-Tuscano et al., 2015; Paulus et al., 2015).

An inhibited young child is likely to stand frozen at the mother’s side with his lips sealed. He will not make eye contact, talk or cooperate at all. It is easy, in these circumstances for the dentist to interpret this as non-compliance or to weigh in with coaxing and a ‘firm hand.’ If interpreted as fear, reassurance will be elicited. These behaviours direct significant levels of intrusive attention toward the child and may be counter-productive (Weinstein et al., 1982; Zhou et al., 2011; Zhou and Humphris, 2013). Chatting to the parent and, almost, ignoring the child gives him a chance to quietly assess the dentist’s demeanour, ‘threat level’ and to evaluate his surroundings.

One early study using non-standard measures found that BI was associated with behavioural signs of distress/non-compliance (Hammock, 1999). Behaviourally inhibited (Radis et al., 1994; Arnrup et al., 2002, 2007) shy, or ‘slow to warm up’ children are at increased risk of isolated dental behaviour management problems (DBMPs) (Quinonez et al., 1997), or DBMPs in combination with dental fear (Klingberg and Broberg, 1998; de Oliveira et al., 2006). Indeed, a proportion (16%) of parents in a study in the Netherlands attributed their child’s dental fear to temperament (ten Berge et al., 2001). Temperament, as measured by the EASI was associated with dental anxiety at 15 years of age (Stenebrand et al., 2013). DBMPs can represent fearful behaviour or non-compliance because of an externalising disorder. However, a vicious circle operates as intrusive parenting results in child externalising problems and reduced effortful control which then provoke intrusive parenting (Eisenberg et al., 2015). Arnrup et al. (2004) found that children with an inhibited temperament retained and increased their risk of being dentally fearful at follow-up, whilst those with an externalising temperament demonstrated an increased risk of non-compliance. Care has to be taken interpreting studies, as the treatment children are expected to accept is sometimes an examination, prophylaxis and fluoride application (Hammock, 1999), or even a filling with a local anaesthetic and the use of rubber dam at a single, often initial visit (O’Callaghan et al., 2006; Allen and Wallace, 2013). These are a significant, potentially overwhelming challenge for young, treatment-naïve children.

Significant correlations were found between 3 aspects of temperament (activity, impulsiveness, and emotional lability) and dental anxiety in 15-year-old adolescents. A regression analysis showed that activity and impulsivity were predictive of dental anxiety (Stenebrand et al., 2013). This is somewhat surprising as these aspects of temperament are associated with approach rather than withdrawal. The distinction between dental fear and dental behaviour management problems (DBMP) is important. Dentists are more likely to identify DBMP than dental fear and children in studies are often referred on basis of DBMP. It is often assumed that DBMPs are the equivalent of dental fear. In one study only 27% children with DBMP had dental fear and 61% of the dentally fearful children had DBMP (Klingberg and Broberg, 2007). This suggests that there is a continuum of uncooperative behaviour in the dental surgery with most cases being mixed fearful and DBMP (ten Berge, 2003). Interestingly, referrals made to HC’s clinic were always on the basis of ‘fear.’ Perhaps this was because parents appeared to find any suggestion that their child is ‘naughty’ very threatening. The effectiveness of treatment of dental anxiety is often assessed using measures of behavioural compliance, although children’s self-report measures are increasingly being used (Fenlon et al., 1993; Kotsanos et al., 2005; Cox et al., 2011).

An interview study in Brazil (de Oliveira et al., 2006, p. 474) found that mothers’ beliefs about their children’s refusal to accept dental treatment were strongly linked to perceptions of

• the children’s temperament (fearful, insecure, shy, dependent, aggressive, responsible, difficult, jealous, uneasy, aggressive, tantrum, perfectionistic)
• a category labelled ‘lack of affection’, which included lack of love and emotionally deprived
• a category labelled ‘protecting children’ which included protection, overindulgence, overprotection, pity, authority and suggests interactions which may be at the level of intrusiveness.

When mothers respond to their child’s fearfulness with intrusive and overprotective behaviours, they are either pushing the child toward a feared, novel stimulus too forcefully or are over-involved in challenges; in ‘helping’ (Kiel and Buss, 2010). This takes control away from the child, minimizing the child’s authentic responses and opportunities to learn how to master unfamiliar situations (Chorpita and Barlow, 1998). These parental strategies may be used with the very best of intentions – to protect their child by discouraging fear reactions which might have negative personal or social consequences, or by protecting the child from experiencing any consequent distress. However, these may increase the risk of undermining healthy emotion regulation development and social interaction (Rubin et al., 2002; Buss and Kiel, 2011). They may be maladaptive for both fearful (Kiel and Buss, 2010) and fearless (Waller et al., 2017) children. Thus, for fearful children, a vicious circle operates, with fearful behaviour triggering intrusion or overprotection and this having long term implications for the development of internalising and externalising behaviours in middle to late childhood (Barrett et al., 1996; Kiel and Buss, 2010).

Interestingly, a large, longitudinal study (Gallitto, 2015) found that, for children with difficult/unadaptable temperaments, exposure to more positive parenting reduced behavioural management problems, demonstrating that positive parenting helps children face challenges. This implies that coaching a parent retains the child’s secure base and provides a modified learning environment for the child and, indeed, a learning environment for the parent. We have found that the dentally fearful parent learns appropriate support and coaching techniques and observes their children develop appropriate and highly functional coping skills. They have reported shifting this learning to other interactions with their children and to taking the skills learned into their own treatment setting. One dentally anxious mother, whose 3 children had been very, very seriously and repeatedly traumatised by their family dentist, told HC, “For various reasons I was not able to change practices, so when I go to the dentist (the one who traumatised the children) and things get difficult for me, I close my eyes and I put myself back here [HC’s clinic] with you and I know I will cope.”

A study with adults found that the emotional lability aspect of temperament was related to dental fear and general psychological distress. However, there were no temperamental differences compared to non-fearful controls (Lundgren et al., 2007). This suggests that temperament and its implications for tolerance of intrusion may play less of a role in dental fear in adults.

Parents with high behavioural inhibition sensitivity (BIS) may overprotect their children to avoid new situations they themselves find threatening. Overprotection may be motivated by concern that something objectively negative could happen (Zelenski and Larsen, 2002) or to avoid a subjectively negative consequence, like their children’s distress, which parents high in BIS may find unpleasant. Overprotection may moderate the link between maternal BIS sensitivity and child internalising behaviour (Kiel and Maack, 2012).

Maternal embarrassment is related to the child’s inhibited behaviour (Kiel and Buss, 2013) suggesting that care should be taken not to further shame or humiliate the mother in the dental surgery, for example by heavy handed feedback about the implications of missing previous appointments.

## Parental Anxiety

Parental psychological control appears to arise from parental separation anxiety, maladaptive perfectionism (Soenens et al., 2006) or hostility toward the child (Pettit and Laird, 2002). The parents are not acting to socialise their children, but to protect their own feelings and needs (Barber and Harmon, 2002). The parent seeks to maintain and defend his/her own psychological status within the family and particularly with the child. This occurs at the child’s expense; the child is not allowed to develop as an individual apart from the parent.

Parental Sensitivity to hurt and disapproval of negative emotion are associated with the use of psychological control (Walling et al., 2007). These parents will be vulnerable to any sense of perceived criticism, so care must be taken when tackling, for example, the subjects of diet and oral hygiene. We have noticed that they also appear to be vulnerable to any *perceived* criticism of their parenting, such as an inference that their child has behavioural problems rather than being scared. Based on clinical observation, we would hypothesise that a fear of being judged as a parent for having a non-cooperative child, has an impact on parent-child interaction under stress.

Inappropriate help with coping may occur because of parental anxiety. Anxious parents are more likely to endorse avoidant coping (Dadds and Barrett, 1996), and are more likely to express fearfulness in front of their children (Muris et al., 1996), possibly as a way of expressing empathy rather than being psychologically intrusive.

During tasks which were relatively unstructured and designed to reflect achievement and social themes, anxious parents of anxious children were more likely to respond with negative behaviours. Non-anxious parents of non-anxious children responded with more warmth. The behaviour of both groups of children mirrored that of their parents, thereby forming feedback loops (Williams et al., 2012).

Parents may interpret ambiguous circumstances as threatening, even when their children do not (Gifford et al., 2008). It may be that anxious parents disambiguate ambiguous situations for their children in an anxiety-fostering manner, thus training their children toward negative appraisals of situations. Children then learn to interpret situations as they would expect their mothers to do so for them (Lester et al., 2010). This may be mediated by information processing biases (Hadwin et al., 2006) such as attention to angry faces (Perez-Olivas et al., 2008). This overinvolvement is a form of over protection. It is the motivation behind the intervention which is crucial to understanding the family dynamic. It is possible to see that this would be a potential mechanism for the links commonly found between maternal and child dental fear (see below).

Anxious children may elicit overprotection which is then exacerbated by parental anxiety (Hudson and Rapee, 2002; Eley et al., 2010). Parental overinvolvement occurs with other, non-anxious, siblings as well as with the anxiety-disordered child (Hudson and Rapee, 2001). Regardless of their own anxiety level, mothers of anxious children are less warm, grant less autonomy and were more likely to catastrophise (Moore P.S. et al., 2004).

Whilst they are capable of accurately predicting, in anxiety provoking situations, the level of ‘challenge’ their children can undertake, parents overestimate the associated levels of distress that children experience and may thus be prompted to use inappropriate coping such as reassurance and overprotection (Cobham and Rapee, 1999). In the dental clinic parents also have a tendency to overestimate their child’s fear, though the parents of highly anxious children underestimated their fear (Krikken et al., 2012b), and the accuracy of such assessments has been called into question (Luoto et al., 2010).

A review (Themessl-Huber et al., 2010) demonstrated that, despite some mixed findings, an association was present between child and parental dental anxiety, though the origins may be various; modelling, informational as well as traumatic conditioning. Since this review, further studies have been published, mostly confirming an association (Olak et al., 2013; Tanaka et al., 2016; Vasiliki et al., 2016; Busato et al., 2017; Shinde and Hegde, 2017).

In the dental clinic parents can be very keen to jump in with information, which is often inaccurate and likely to foster anxiety. However, it is our experience that most parents do this only when they believe their child is not getting enough information. If the dentist truly is providing enough information to alleviate the child’s anxiety (and for some children, ‘less is more’) then this needs to be pointed out very carefully to the parent. Information should be combined with a stop signal and subjective units of distress scale (SUDS) to give an overt indicator of fear level and need for behavioural and informational control (Chapman and Kirby-Turner, 2002). This can then be pointed out to the mother. This process tends to contain maternal catastrophising. Similarly, the mother who, on the way to the appointment, answers an anxious child’s questions with information that reflects her own fears, can be contained by suggesting that the child has a notebook in which ‘Mummy helps you write down your question so that you don’t forget. Then the dentist can tell you what you want to know when you get there.’

A classic example of an overinvolvement and over protectiveness is that mother who, on bringing her toddler into the dental clinic for the first time for an examination says, “She’s [the dentist’s] not going to hurt you,” intending to reassure, but, instead, raising the possibility that the child will be hurt. This has been assessed as a problem with invasive medical procedures (McMurtry et al., 2010). However, some intrusive parenting witnessed in the dental clinic may not reflect the home situation; an anxious mother under pressure may display a different parenting style to the one she habitually uses. This may be because she is struggling to contain her own, situationally triggered anxieties, or because she fears being judged or shamed as an inadequate parent for ‘not being able to support or control’ her child in the clinic or for allowing them to develop dental decay.

Parental self-reports of parenting interactions differ from those observed in studies (Bennetts et al., 2016). Parents under pressure, such as those taking a frightened child to the dentist, theoretically may behave in subtly different ways to their normal behaviour, although the authors are unaware of any relevant literature. Care has to be taken with labelling parental behaviour as unhelpful as, in stressful situations, *well-intentioned* parental ‘helpful’ coping such as reassurance, criticism, apology, giving control to the child and empathy can backfire and promote distress in medical situations (Blount et al., 2001; Salmon and Pereira, 2002).

The children of depressed parents are more likely to develop internalising and externalising problems. In boys, but not girls, this is associated with intrusive parenting (Gruhn et al., 2016). As dentists, we often see whole families; parents will disclose their medical history. We should be mindful that current and previous parental depression may impact on the children’s ability to accept treatment.

Dentists often [more frequently in North American than in Europe (Marcum et al., 1995; Crossley and Joshi, 2002)] prefer to exclude parents on the basis that children behave better, that is are more compliant, when seen on their own (Marzo et al., 2003; Cox et al., 2011). For only mildly anxious children, it may be that, in the absence of the mother, they inhibit protest and thus appear to be fearless and more easily treated (Shaw and Routh, 1982). However, other stated reasons include that it wastes time and makes the dentist uncomfortable (Marcum et al., 1995). It is tempting to hypothesise that dentists themselves may find anxious, over-protective mothers psychologically intrusive; a threat to their sense of professional autonomy and sense of control. This has been established as an important factor in stress-related practise for dentists (Chapman et al., 2015). However, there are increasing parental requests to remain in the clinic (Shroff et al., 2015) and this is associated with greater levels of parental satisfaction with their child’s visit (Kim et al., 2012).

## Effects on the Individual

Most of the research to date has been conducted on adolescents as it is believed that the greatest effects are seen within this age group. However, younger age groups are now being investigated using observational studies and children as young as 9 years old can report aspects of psychological control by their parents. We believe that aspects of parental behaviour may be apparent in clinical situations at an even younger age. The impact of childhood intrusive parenting on adults has received little attention in comparison.

Intrusive parenting serves to limit the child’s self-expression, define his emotional experience and inhibit his thinking processes (Barber, 2002). The child may be harmed in a number of ways:

• It limits free decision-making, self-expression and self-discovery.
• It controls self-will and reduces self-reliance, self-confidence, self-esteem, self-efficacy and self-regulation of emotions, all of which have implications for the ability to accept dental treatment.
• This interferes with psychosocial maturity (individuation, development of identity and individuality, independence, emotional autonomy) which usually helps to develop a sense of boundary between the self and the outside world. These boundaries may be culturally sensitive (Nucci and Smetana, 1996; Barber, 2002).

As well as the wealth of cross-sectional data for the detrimental effects of psychological control on children, longitudinal research (with children from the age of 5–6 years old to 7–8 years old) has found that maternal, but not paternal, psychological control, in combination with high levels affection, predicts levels of internalising and externalising disorders. Behavioural control, in combination with low levels of psychological control, decreased externalising problems (Aunola and Nurmi, 2005).

When separated from their parents and faced with, even routine tasks which are novel to them, children who are infantilized and have low self-efficacy suffer an increase in anxiety levels. This in turn causes distress and thus impairs learning and skills acquisition (Wood, 2006).

Psychological control (over-protection, lack of warmth and intrusiveness) appears to be related to the later childhood development of internalizing problems; withdrawn behaviour, passive resistance, eating disorders, suicidal ideation, somatic symptoms, simple phobias, separation anxiety disorder, depression, (though not generalised anxiety disorder) in cross-sectional (Arrindell et al., 1983; Gerlsma et al., 1990; Barber et al., 1994; Barber, 1996; Overbeek et al., 2004; Wood, 2006; Wood et al., 2007; Reitman and Asseff, 2010) and longitudinal studies (Enns et al., 2002). However, there are less consistent associations with externalising problems (aggression, antisocial behaviour, defiance and, most commonly, delinquency) (Barber et al., 1994; Barber, 1996; Barber and Harmon, 2002; Pettit and Laird, 2002). Overparenting is reported to have similar outcomes for children (Gar and Hudson, 2008). This association continues into emerging adulthood (Desjardins and Leadbeater, 2017).

Depending on the particular nature of parents’ psychological control, emerging adults reported both higher, and inappropriate, levels of dependence on parents with implications for emerging adults’ feelings about the upcoming transition to adulthood (Lindell et al., 2017). As young adults they have an increased sense of entitlement and higher levels of narcissism, believing that others can, and perhaps should, solve their problems and provide assistance and support. This is associated with ineffective coping skills (Segrin et al., 2012, 2013). It is theoretically possible that adult dental patients who are quick to be angry, rather than display anxiety (Linehan, 1993), have had this type of upbringing. The patient who demands appointment exactly when convenient to them, with no allowances for the complexities of the appointment book; their job, unlike other people’s is far too important to miss.

It may be that there are differential effects depending on gender, with only female anxiety being associated with maternal control and paternal acceptance (Reitman and Asseff, 2010).

Helicopter parenting in childhood is associated, in college students, with reduced engagement with education (Padilla-Walker and Nelson, 2012), higher levels of depression, less satisfaction with family life (Givertz and Segrin, 2014; Schiffrin et al., 2014), increased use of prescription medication to treat anxiety and depression and with the recreational consumption of pain pills (LeMoyne and Buchanan, 2011). The students’ reduced well-being is largely associated with reduced autonomy granting and perceived competence (Barnett et al., 2010; Schiffrin et al., 2014).

Ironically, helicopter parenting is also associated with positive aspects of parenting such as involvement, guidance, warmth, disclosure and emotional support (Padilla-Walker and Nelson, 2012). It does seem to hinder the development of adult independence (Lindell et al., 2017).

Parental over-involvement can lead to low self-esteem in adults (Winefield et al., 1990). Middle-aged adults who had been subject to parental control as children were found to have more problems with interpersonal relationships suggesting that interpersonal competence in childhood is an important influence on adult mental health (Rodgers, 1996a,b). This pattern may be influenced by the early maladaptive schemas developed as a result of intrusion; mistrust, shame, fear of abandonment. It persists into adult life and impacts on interpersonal relationships (Messman-Moore and Coates, 2007) and is associated with depression (Ono et al., 2017).

Adults whose parenting has been overinvolved or ‘helicopter’ show lower levels of self-efficacy (Givertz and Segrin, 2014; Darlow et al., 2017). The reduction in self-efficacy has been found to be associated with anxiety, depression, life satisfaction and physical health (Reed et al., 2016). Helicopter parenting has to be carefully distinguished from the related construct of ‘autonomy supporting’ which is directly related to life satisfaction and physical health (Reed et al., 2016). Low levels of self-efficacy have implications for preventive dental behaviours (Kakudate et al., 2010) and dental fear is linked to low self-efficacy in adults (Kent and Gibbons, 1987). It is therefore theoretically possible that self-efficacy mediates an association between intrusive parenting and child and adult dental fear.

Gelotophobia is the fear of being laughed at. This can be learned vicariously from parents, but is also associated with parental psychological control in childhood (Proyer et al., 2012) and, presumably, the threat of humiliation and being shamed.

Stalker et al. (2005) highlight the fact that victims of childhood sexual abuse (CSA) may be particularly vulnerable to perceived judgement and criticism. These patients have increased levels of dental fear (Willumsen, 2001, 2004). Many may have developed mental schema or beliefs about themselves as undeserving or bad, rendering ambiguous comments or ill-advised attempts at humour liable to be interpreted to fit these mental models of the self.

There is evidence that eating disorders may be associated with intrusive parenting (Rorty et al., 1994, 2000; Soenens et al., 2008; Ketisch et al., 2014) and some evidence that dental fear levels are raised in this group (Sirin et al., 2011). This suggests that when treating known and about-to-be-diagnosed (for example, the dentist notices severe palatal erosion) patients with eating disorders, dentists need to be mindful of the patients’ increased vulnerability to intrusion – putdowns, sarcasm, belittlement and perceived criticism.

We hypothesise that this learned model, which is internalised during childhood, can continue to function through childhood and into adulthood. It may be that a well-intentioned dentist can come to represent the intrusive parent. The balance between the concerned professional persisting in giving advice regarding diet and oral hygiene techniques may fall into this role. The quote below is from a dentist in a previous study (Chapman et al., 2015) who had seen that continual encouragement/nagging could be counterproductive.

“The patient who isn’t responding to your suggestions of tooth brushing, well, you still have to try to; you shouldn’t give up on them, even if they don’t respond. You’ve still got to try to motivate them; to get them to see why you’re telling them these things. Or else you just agree with them to disagree and warn them of the down sides of not complying with you. Effectively you say, look, I’ve told you how to do this; shown you. If you don’t, your mouth’s going to suffer, but if you’d rather I didn’t nag every time, let’s just agree that this is how things are and that you know that it would be the best for you that you do it.”

## Psychological Intrusion in Dentistry – Evidence From the Literature

We have previously argued that psychological intrusion can be a problem for patients in the dental clinic (Chapman and Kirby-Turner, 1999; Chapman and Kirby-Turner, 2005). There are fewer indications that psychological intrusion has an impact on dental fear than for physical intrusion, but they are present. A summary of questionnaire items which might indicate a fear of psychological intrusion is presented in **Table 1**.

**Table 1:** Examples of items from various questionnaires revealing possible dental psychological intrusion.

Study	Child/adult	Questionnaire	Item
Al-Namankany et al., 2012a	Child	The Abeer Children Dental Anxiety Scale	Do you feel shy at the dentist? Do you feel shy because of the way your teeth look? Are you worried about losing control at the dentist?
Welly et al., 2012	Child	Untitled questionnaire	I would feel better if the dentist doesn’t speak to me. My dentist treats me like a baby. That is comfortable. My dentist speaks with me too much.
Gale, 1972	Adult	Untitled questionnaire	Dentist laughs as he looks in your mouth.
Scott et al., 1984	Adult	Untitled questionnaire	To what extent have you felt reluctant about talking with your dentist(s) about your possible dental anxiety? To what extent have you been fearful of a reprimand from your dentist(s) due to your possible poor oral hygiene? To what extent have you been criticised by others for being anxious or afraid of dental treatment? To what extent have you felt ashamed of your dental fears and anxieties?
de Jongh and Stouthard, 1993	Adult	Untitled questionnaire re hygienist treatment	The dental hygienist not being nice. Remarks about poor oral hygiene.
de Jongh et al., 1995	Adult	Dental Cognitions Questionnaire	Dentists think you act childish. The dentist believes that I am a difficult patient and act childish. I should be ashamed of my teeth.
Moore and Brødsgaard, 1995	Adult	Semi-structured interview – themes	A feeling of powerlessness/embarrassment. The things dentists did or said that made things worse, e.g., condescending remarks/rejection Powerless/dominant dentist.
Milgrom et al., 1995	Adult	Dental Beliefs Survey	I believe dentists do/say things to withhold information from me. I feel uncomfortable asking questions. I am concerned that dentists will not take my worries (fears) about dentistry seriously. I am concerned that dentists will put me down (make light of my fears). I am concerned that dental personnel will embarrass me over the condition of my teeth. I am concerned that dentists do not like it when a patient makes a request.
Coolidge et al., 2005	Adult	Dental Beliefs Survey-Revised	I feel uncomfortable asking questions. I am concerned that dentists will not take my worries (fears) about dentistry seriously. I am concerned that dentists will put me down (make light of my fears). I am concerned that dental personnel will embarrass me over the condition of my teeth. Dental professionals say things to make me feel guilty about the way I care for my teeth. I am concerned that dentists do not like it when a patient makes a request. I believe that dentists don’t have enough empathy for what it is really like to be a patient.
Stouthard et al., 1995	Adult	Dental Anxiety Inventory (DAI)	I already feel uncomfortable at home when I think that the dentist will make a remark about my teeth. On my way to the dentist, I sweat or freeze at the thought that the dentist will say I brush my teeth badly. I feel uncertain when discussing the treatment of my teeth with the dentist. Before going to the dentist, I get palpitations when I think of how the dentist will be displeased at my teeth. When I am on my way to the dentist and think that she/he will say my teeth look bad, then I want to go home again. In the waiting room, I feel nervous at the thought that the dentist will say my teeth are badly brushed. I think about cancelling the appointment if I suspect the dentist will be displeased at my teeth.
Aartman and Hoogstraten, 1999	Adult	Social Attributes of Dental Anxiety Scale (SADAS)	I have arguments with friends, partner or parents about visiting a dentist. I feel that people put pressure on me to visit a dentist. I feel that no one shares my fears about visiting a dentist. I feel that people will laugh at me if I tell them of my fears of dentistry.
Abrahamsson et al., 2002	Adult	Unstructured Interviews – themes	Feelings of powerlessness (Total lack of control about what happens; The dentist has the power). Feelings of being deserted and vulnerable (feelings of shame for being childish or behaving badly). Unsupportive dentist (The dentist did not listen to my signals).
Locker, 2003	Adult	Psychosocial Consequences Scale	I feel foolish being afraid of dental treatment. I feel people will laugh if I tell them about my fears of dental treatment. I have arguments with friends, partner or family about not going to the dentist. People tell me my fears of dental treatment are childish or ridiculous. I get annoyed when people try to pressure me into having dental treatment.
Oosterink et al., 2008	Adult	67 Item Questionnaire	A remark made by the dentist Dentist’s manner.
Stenman et al., 2009	Adult	Unstructured interview themes	The need to be treated respectfully Feelings of control over the situation.
Armfield, 2010	Adult	IDAF-4C	To what extent are you anxious about the following things when you go to the dentist? Feeling embarrassed or ashamed Having an unsympathetic or unkind dentist.

In questionnaire measures used with older adolescents and adults a number of items suggest an element of psychological intrusion, though it is not possible to definitely assert this without a deeper understanding of the meaning of the question/fear to the patients concerned. For example, the item, “The dental hygienist not being nice,” (de Jongh and Stouthard, 1993) could mean different things to different people; Does the hygienist put you down? Is s/he generally grumpy? Does s/he nag?

Threats from intrusion to the definition of the self are often presented as threats to self-esteem within the dental literature (Stouthard and Hoogstraten, 1987; Chapman and Kirby-Turner, 1999, 2005). Poor self-esteem is associated with dental fear/anxiety (Stouthard and Hoogstraten, 1987; Schuurs et al., 1988; Kvale et al., 2002; Locker, 2003). Significant differences were found in the answers to questions (which could be interpreted as measuring threats to self-esteem) between fearful and non-fearful students (Scott et al., 1984). Success in accepting dental treatment is associated with an increase in reported self-esteem (Kvale et al., 2002).

Shame has been found to be related to dental anxiety (de Jongh et al., 1995; Abrahamsson et al., 2002; Armfield, 2010). In the dental literature this appears, sometimes, to be labelled as embarrassment (Moore R. et al., 2004) and is the result of ‘put downs’ (Moore et al., 1993). Later research (Moore et al., 1996) found that a history of putdowns was reported by 34.2% of Americans (and was significantly associated with avoidance) and 11.7% of Taiwanese. This implies a cultural element to this psychological threat to the sense of self as suggested by Nucci and Smetana (1996).

Belittlement forms part of the Dental Beliefs Survey (DBS) (Milgrom et al., 1995) and explains a significant proportion of dental fear; indeed Johansson and Berggren (1992) found that the most strongly correlated relationship was between dental fear and the belittlement subscale (‘Dentists make me feel guilty, Dentists don’t take my worries seriously. I worry what dentists think of me.’) of the DBS. It is interesting to note that although the original version of the Dental Beliefs Scale had a belittlement subscale, this was lost in the Revised Scale (Milgrom et al., 1995; Coolidge et al., 2005).

Moore et al. (1993) also noted a strong relationship between dental fear and the dentist being angry. We don’t know the meaning of this for the patients, but it may relate to humiliation, loss of control or fear of a more ‘powerful’ other; the angry dentist may be subconsciously representing the critical and/or angry parent (Linehan, 1993).

Intrusions often result from humiliation, sometimes as result of an unhelpful attempt at the use of humour, by the dentist, to lighten the atmosphere. This is reflected in items in **Table 1**.

The possible, differing interpretations of these items may be reflected in a change in importance of particular items in cross-sectional study. For example, ‘Dentist laughs while he looks in your mouth’ was found to be highly anxiety provoking by Gale (1972) but much less so in a later study (Stouthard and Hoogstraten, 1987). However, it is consistent with the development of gelotophobia as a result of intrusive parenting (Proyer et al., 2012).

The literature considering the effectiveness of the dental team behaviour on child anxiety relies largely on behavioural measures which are effectively measuring non/cooperation that may be associated with fear or dental non-compliance (DBMP), (Klingberg, 2008) but are often deemed to represent the absence of fear. Without understanding the meaning of the observed events for the children concerned, we propose that the following observations may reflect the impact of psychological intrusion by the dentist or team on child patients; putdowns, coaxing, coercion, (Weinstein et al., 1982) and punishment and criticism (Melamed et al., 1983). Zhou et al. (2012b) found that extended duty nurses (EDDNs) were more successful at completing fluoride applications when they used praise, instruction, and information-giving to guide the children’s behaviour compared to permission seeking, offering alternative tasks, information seeking and reassurance. However, a review of the literature suggests that findings from earlier research were inconsistent (Zhou et al., 2011). Children with externalizing behaviour (possibly reflected in the dental clinic as non-compliant DBMPs) may elicit intrusive mothering as a way of helping the children learn to self-regulate behaviour (Eisenberg et al., 2015). The detailed research examining clinician-child interactions (Weinstein et al., 1982; ten Berge et al., 1999; Zhou et al., 2012a) could be extended to examine the tripartite dentist-parent-child relationship for potentially intrusive adult behaviour.

In a study of treatment of highly fearful children, they were treated in a more authoritative and controlling way than lower fear children (ten Berge et al., 1999). Children who were more fearful were subject to significantly more ‘put downs,’ had their expressed feelings denied or ignored and were subject to significantly more physical restraint. They were also subject to more (non-significant) levels of coercion and coaxing. If an element of these children’s fears was based on intrusive parenting, this study suggests that fear may elicit intrusive behaviours from the dentists treating them. This would represent a vicious circle. The more fearful children improved more in the course of treatment than the non-compliant. However, given the relationship between externalising disorders and psychological intrusion, it is theoretically possible that fear of intrusion may also have been active in the non-compliant children with DBMPs. Traditional measures of child fearful behaviour are unlikely to identify the child who is afraid of intrusion by the dentist.

In a review, all identified self-report dental fear/anxiety questionnaires for children were found to rely on fear of situations/treatment (Al-Namankany et al., 2012b). The Abeer Children Dental Anxiety Scale (Al-Namankany et al., 2012a) was developed to contain a cognitive element, with questions apparently relating to embarrassment/shame – ‘Do you feel shy because of the way your teeth look?’ – and a need for control, but which might relate to intrusion. A word of caution is needed regarding the item, ‘Are you worried about losing control at the dentist?’ the meaning of this is ambiguous; does the child fear panicking or does the child fear not being able to control what is happening to him/her? Also, it is doubtful that many 6-year-olds would comprehend either meaning of this question. However, the questionnaire is validated with primary school children age 6–11. Another, untitled questionnaire (Welly et al., 2012), contains several items which may well represent intrusion (**Table 1**).

The boundaries between positive hypnotic suggestion and intrusive behaviour by the dentist are not at all obvious. Peretz (1996) gives a detailed description of the treatment of an anxious 11 year old boy. He describes structuring part of the conversation to end with four statements

“to which I was sure the boy would agree. (1) ‘I understand that you were in a few doctor’s offices before.’ (2) ‘I know you are afraid now.’ (3) ‘I understand that you are having pain in your teeth.’ And (4) ‘I see you want your teeth to be treated, but also, not to be so afraid”’ (p. 206).

Most children would accept these statements in the spirit in which they were intended; to convey understanding of his fear, predicament and wishes. For an individual who has been subject to psychological intrusion, these statements may have been interpreted very differently; as *telling* him how he is feeling and what he *is* thinking, thus removing any control of his own thought processes. This can be unsettling or frankly traumatic.

It raises the issue as to whether an individual who is vulnerable to psychological intrusion would actually present as hypnotisable or whether the fear of losing control over one’s own beliefs and thought processes might render them ‘unhypnotisable’ as a form of self-protection.

## Implications for Dental Care

We have previously suggested that it is difficult to directly treat dental fear based in a fear of intrusion and that the problem has to be tackled by addressing other elements of the Chapman and Kirby-Turner model of dental fear (Chapman and Kirby-Turner, 1999, 2005). Children who are currently, and adults who have been, subject to psychological intrusion, may seize any opportunity to take whatever control of a situation they are offered or can take advantage of. This is probably the most important aspect of the model to address, though fear of betrayal, pain and the unknown are also important.

Control is the ability to personally influence events and outcomes in one’s environment, principally those related to positive or negative reinforcement (Chorpita and Barlow, 1998). When working with children, the degree of control shared with the child must be age appropriate. Control can be achieved through: behaviour (direct action on the environment), regulated administration, stimulus modification, cognitive, information gain, appraisal, and decisional control (having a choice among alternative courses of action) (Averill, 1973). There is a difference to be highlighted between experienced and objective control, both of which are achieved through decisional control (Law et al., 1994). Age appropriate objective control for a 3- year-old might be the choice between different coloured protective glasses, or whether to have the upper right teeth or the upper left teeth cleaned first with the electric toothbrush. For an adult, experienced control and objective control should be equivalent. In between there should be a sliding continuum along which experienced and objective control approximate.

A case we treated involved a needle phobic teenage girl who was subject to significant psychological control at home. She had previously, aged 8, had treatment under general anaesthetic and had been offered desensitisation immediately afterward. Despite being told that this was a routine procedure for children at this age, her mother had refused on the grounds that she was not old enough to manage it. (This was interpreted as over-protection and infantilization to maintain intrusive control.) Seven years later she needed a restoration replaced and she, herself, refused another general anaesthetic or sedation. HC took her through a CBT-based desensitization programme. At each stage, she initially refused to do the task which had previously been agreed upon; at each stage HC worked through the situation with her and pointed out that, on every previous occasion, she had eventually managed to complete the task. Eventually this stopped being effective and the girl explained that she felt that HC was over-riding her sense of control and acting rather like a ‘snake charmer’ and manipulating her to do things against her better judgement. Most young people would have seen such persuasion as a demonstration of faith in their ability to cope and complete the task, but, for this girl, it was an act of profound psychological intrusion. The impasse was eventually broken by HC giving her supervised control of a dental probe (explorer) to prod herself, eventually quite hard, to check, for herself, that the topical anaesthetic had worked.

Intrusive relationships are particularly difficult to deal with within the surgery. If subject to intrusive parenting, young children will have reduced self-efficacy. The child is likely to avoid situations of enforced separation from parents as they do not believe they can manage alone. Because they are not used to coping and problem solving on their own, they may be far more likely to catastrophically misinterpret ambiguous situations so care needs to be taken to shift at a slow enough pace, with sufficient explanations, to alleviate the child’s anxiety. On the other hand, the presence of the parent provides safety and reassurance thereby reducing child anxiety and negatively reinforcing the child (Bowlby, 1988). (Negatively reinforcing because of the removal of anxiety.) Thus, the child is likely to want to avoid a situation of enforced separation from parents and refusal to allow an intrusive parent to accompany their child, or prompt removal of an intrusive parent, is likely to provoke heightened child anxiety (see below).

We have found that the way to deal with this is to build a relationship with the child over several visits, during which the parent can witness that everything is explained to the child and that their *own* perceived ability to cope is monitored very carefully using a rating scale (Chapman and Kirby-Turner, 2002). During this induction phase of overtly non-threating treatment, such as a prophylaxis brush on the finger and then the teeth, the parent may well keep interrupting to check that the child is all right; “Are you *sure* you’re alright?” the implicit message to the child is, “Maybe you’re not OK.” However, it can then be pointed out to the parent that the child has a stop signal and hasn’t used it. The child can be asked if they needed to use the signal and ‘forgot’ to do so. Once a good relationship with the child is established, one of two outcomes are possible. Either the parent has relaxed and is far less anxious about the situation, is no longer trying to control it and is a help in the surgery – s/he has learned some coping skills, or the parent is still ‘interfering.’ In the latter situation, the dental nurse, at the next visit, requests the child to come by name from the waiting room, the child gets up spontaneously and makes no gesture to request the parent to accompany them, the parent can be encouraged to remain, with the caveat that if the child changes his/her mind, or the treatment plan changes, the parent will be invited into the surgery. The child may need to be prompted by a simple enquiry as to whether they would like to come on their own. The same caveats apply. This represents a confident child making *age appropriate* choices and who is separated from the parent without offence.

Despite being in obvious distress, some children are so reluctant to use a stop signal and make a control request, that they fail to use it. In these circumstances they have to be coached in its use. They can be instructed to request something ‘silly’ like needing to scratch their noses. Having practised this, they can then rehearse asking for a rest, before they need it. We hypothesis that this may be associated with a history of psychological control and/or behavioural inhibition.

The parent may behave in other unhelpful ways, but rather than ask them to leave, a tripartite system of working with the parent which fosters child independence and encourages the parent to ‘leave the child alone.’ For example, if the parent is touching too much toward the trunk, hands and face, they can be asked to sit at the foot of the dental chair and hold/stroke the child’s ankle. This tends to move the parent out of the child’s most intimate personal space and de-escalates the overprotectiveness of the physical contact.

The parent who keeps telling the child, presumably as a way to reassure, “It’s nearly finished,” when one has only just started, can be contained by explaining, in detail, with a guided tour of the cavity (including, via a mirror, the child if s/he would like to be), exactly why the filling is not nearly finished. The parent then can then be encouraged to look regularly and learns to give accurate feedback on how much better things are looking and sounding (a way of differentiating healthy from unhealthy dentine). They can become excited as progress occurs and this positive message of progress is helpful.

Because they are not used to coping and problem solving on their own, children who are psychologically controlled may be far more likely to catastrophically misinterpret ambiguous situations. False reassurance such as solicitous inquiries such as, “Are you OK?” “Are you *sure* it’s not hurting?” which occur despite a structured stop signal-fear thermometer combination being in place and not being used by the child, (Chapman and Kirby-Turner, 2002) only serve to increase fear. This can be gently addressed by acknowledging that the parent (usually mother) is only trying to ensure that the child is OK, but that her child’s experiences are different from those she had; that the child has had every opportunity to let the dentist know that s/he is not coping and has chosen not to do so. The child is than asked to confirm that s/he really would let the dentist know if there were a problem. This defuses the situation and allows the parent to stay with the child to provide a secure base; the presence of the parent provides safety and reassurance thereby reducing child anxiety and negatively reinforcing the child by of removal of anxiety (Bowlby, 1988).

Including the parent, wherever possible, in cognitive-behavioural type treatment of dental fear is consistent with the involvement of parent in the treatment of children with anxiety disorders (FCBT) and can lead to better outcomes (Creswell and Cartwright-Hatton, 2010; Hudson et al., 2014; Manassis et al., 2014). Indeed, there is some evidence that effectiveness of FCBT in treating anxiety disorders in children is mediated by a reduction in parental intrusiveness (Wood et al., 2006, 2009).

Some teenagers may feel that their parents are ‘interfering’ and may be heartily relieved to accede to a suggestion that they might like to come into the surgery alone. It is, however, essential that, as with younger children, the parent does not feel completely excluded or alienated.

Adults who have been subject to intrusive parenting are going to be particularly vulnerable to any situation in which they perceive that they are being laughed at, even if they are not diagnosed with gelotophobia. It behoves dentists, when using humour, to ensure that they are not perceived to be laughing *at* their patients. They are likely to need a high degree of control over their treatment and to be vulnerable to fear of betrayal. Recent reviews found that CBT and behaviour therapy had the most evidence of efficacy in the treatment of dental fear in adults (Gordon et al., 2013; Heaton, 2013), though even with this, care needs to be taken in the way patients are encouraged to challenge their negative thinking patterns as too great an enthusiasm in ‘helping’ with challenging negative automatic thoughts on the part of the dentist or therapist is open to interpretation as intrusion.

Finally, and no doubt controversially, we have to consider what is possibly the ultimate intrusion by dentists on child patients. A large variety of ‘child behaviour management’ techniques have been researched for effectiveness in helping children accept treatment, be that in terms of over-coming non-compliance or fear, the two often being confused (Klingberg, 2008). One of these is ‘escape and reward’ or contingent escape. There are variations of this procedure. The ‘poorly behaving’ child patient may be told that a procedure, be that in an introductory session (Allen and Stokes, 1987; Allen et al., 1988) or active treatment such as a prophylaxis or drilling (Allen et al., 1992), will continue until s/he behaves properly and then s/he will be given a rest. In another variation, the child is given regular breaks from treatment on a fixed time-schedule, independent of the child’s behaviour (O’Callaghan et al., 2006). This technique appears to be well accepted by parents (Elango et al., 2012). A more recent version of the technique is parental presence/absence or PPA; poorly behaved, uncooperative children are ‘empathically offered’ parental presence if they are cooperative (Molinari and DeYoung, 2004; Kotsanos et al., 2005, 2009). These techniques do appear to allow dentists to complete more clinical work, but at what price to the child’s mental well-being?

## Conclusion

We believe that psychological intrusion plays an important role in the aetiology of some children’s dental fear and continues to affect individuals into adulthood. Belittlement and shaming experiences may be particularly salient. However, the relationships between child dental anxiety, child temperament (behavioural inhibition), parental (particularly maternal) dental anxiety and general anxiety, parenting style and intrusive parenting are complex.

Dentists may inadvertently act intrusively toward patients with negative consequences.

There is a need for detailed, good quality research to explore the extent and exact nature of these problems. The development of a simple, non-threatening means of helping dentists identify patients for whom psychological intrusion is a problem would be helpful.

## Author Contributions

HC wrote the first draft and constructed the diagrams. NK-T was responsible for the original clinical observations and development of the idea. He contributed to and reviewed the content of the article and illustrations.

## Conflict of Interest Statement

The authors declare that the research was conducted in the absence of any commercial or financial relationships that could be construed as a potential conflict of interest.
